# Behavioural Analyses of Quinine Processing in Choice, Feeding and Learning of Larval *Drosophila*

**DOI:** 10.1371/journal.pone.0040525

**Published:** 2012-07-10

**Authors:** Amira El-Keredy, Michael Schleyer, Christian König, Aslihan Ekim, Bertram Gerber

**Affiliations:** 1 Genetics and Neurobiology, University of Würzburg, Würzburg, Germany; 2 Genetics, Tanta University, Tanta, Egypt; 3 Genetics, University of Leipzig, Leipzig, Germany; 4 Molecular Biology and Genetics, Bilkent University, Ankara, Turkey; 5 Genetics of Learning and Memory, Leibniz Institute for Neurobiology, Magdeburg, Germany; 6 Behavioural Genetics, University of Magdeburg, Magdeburg, Germany; AgroParisTech, France

## Abstract

Gustatory stimuli can support both immediate reflexive behaviour, such as choice and feeding, and can drive internal reinforcement in associative learning. For larval *Drosophila*, we here provide a first systematic behavioural analysis of these functions with respect to quinine as a study case of a substance which humans report as “tasting bitter”. We describe the dose-effect functions for these different kinds of behaviour and find that a half-maximal effect of quinine to suppress feeding needs substantially higher quinine concentrations (2.0 mM) than is the case for internal reinforcement (0.6 mM). Interestingly, in previous studies (Niewalda et al. 2008, Schipanski et al 2008) we had found the reverse for sodium chloride and fructose/sucrose, such that dose-effect functions for those tastants were shifted towards lower concentrations for feeding as compared to reinforcement, arguing that the differences in dose-effect function between these behaviours do not reflect artefacts of the types of assay used. The current results regarding quinine thus provide a starting point to investigate how the gustatory system is organized on the cellular and/or molecular level to result in different behavioural tuning curves towards a bitter tastant.

## Introduction

The sense of taste is that component of the contact chemosensory system devoted to organize feeding, allowing animals to prefer edible and avoid toxic substances. In addition, gustatory stimuli can be reinforcers: They can induce memories for stimuli or actions that preceded them, such that the animal can find good and avoid bad food. Gustatory stimuli thus organize both immediate, reflexive behaviour towards food (such as choice and feeding), and, by virtue of their association with predictive stimuli or instrumental actions, the search for food. Trivially, these functions must come about by different sets of neurons on at least some level of processing. While at the level of gustatory interneurons such dissociation can clearly be found (e.g. in terms of the sufficiency of octopaminergic signalling for reinforcement, but not for ingestive behaviour [1]–[3]), it is not resolved in detail whether and how different sets of sensory neurons organize different gustatory reflex behaviours and/ or internal reinforcement signals, respectively (for an interesting study of this issue in mice see [4]). Here, we want to take a first systematic step into such an analysis by behaviourally “footprinting” the dose-effect characteristics of bitter-processing (“bitter” is used throughout this study in the sense that humans verbalize these chemically diverse and often toxic substances as “tasting bitter” and avoid eating them) in choice, feeding and reinforcement processing of larval *Drosophila*, using quinine as a study case.

**Figure 1:**
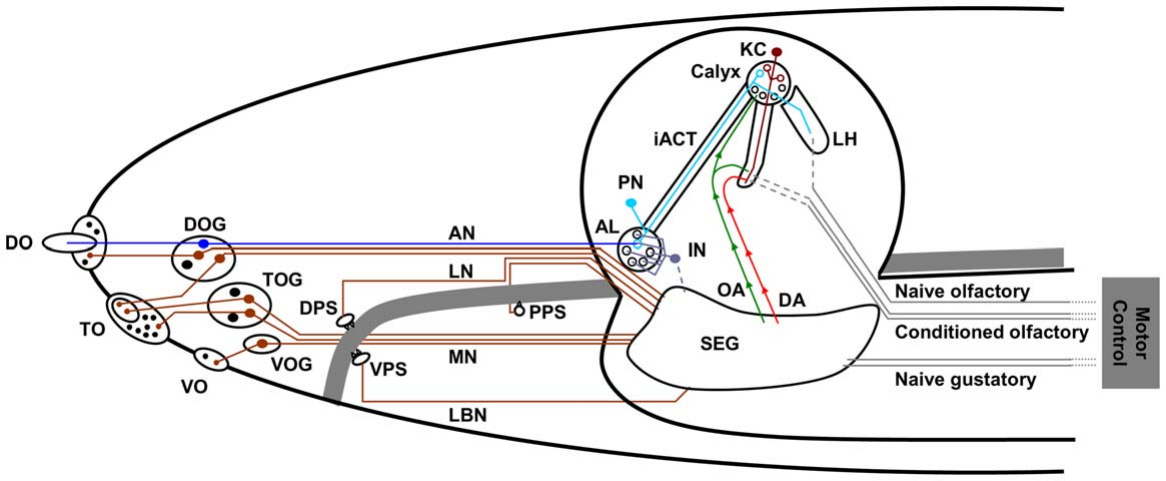
The anatomy of the *Drosophila* chemosensory system. Overview of the cephalic larval chemosensory pathways. Olfactory pathways (blue) project into the brain *proper*, whereas gustatory afferents (brown) are collected in various regions of the subesophageal ganglion. The green and red arrows indicate pathways to short-circuit a taste-driven reinforcement signal from the subesophageal ganglion towards the brain. Note that the different gustatory organs project to different regions in the subesophageal ganglion. Abbreviations: AL: antennal lobe; AN: antennal nerve; DA: dopaminergic neurons as engaged in reinforcement signalling; DO/ DOG: dorsal organ/ dorsal organ ganglion; DPS: dorsal pharyngeal sensillae; iACT: inner antenno-cerebral tract; IN: antennal lobe interneurons; KC: Kenyon cells; LBN: labial nerve; LH: lateral horn; LN: labral nerve; MN: maxillary nerve; OA: octopaminergic neurons as engaged in reinforcement signalling; PN: projection neurons; PPS: posterior pharyngeal sensillae; SEG: subesophageal ganglion; TO/ TOG: terminal organ/ terminal organ ganglion; VO/ VOG: ventral organ/ganglion; VPS: ventral pharyngeal sensillae. Based on Stocker 2008 [10].

The larva is the growth and feeding stage of the *Drosophila* life cycle and as such is a suitable study case for taste research. Substrate choice, feeding and reinforcement learning can be tackled by simple, cheap and well-defined behavioural assays; in addition, the larval gustatory system is comprised of relatively few neurons and is beginning to be described at the anatomical, cellular and to some extent also the molecular level [5]–[7] (for reviews with emphasis on the larva see [8]–[12]; for more general reviews on the neurogenetics of chemosensation see [13]–[18]). In principle, the cellular architecture of taste processing seems to conform to what had been found in adults (see reviews cited above) (Fig. 1). However, the exact relation between cellular identity, expression of putative gustatory receptor molecules from the *Gr-* [19] and/ or *Ir*-family of genes [20], their molecular mode of action, their ligand profile, the terminal projection patterns of their host gustatory sensory neurons and their behavioural roles are far from being satisfyingly clear (see Discussion). To take a first systematic step into an analysis of larval bitter-processing, we parametrically describe at the behavioural level the effects of various concentrations of quinine hemisulfate. Specifically, we examine the following questions:

- How does quinine concentration affect choice between bitter and tasteless substrates?
- How does quinine concentration affect feeding behaviour?
- How do different quinine concentrations differ in their reinforcing power?
- How do the respective dose-effect curves relate?

These experiments, we hope, will provide a framework to investigate how the gustatory system is “orchestrated” on the cellular and/ or molecular level to support different kinds of behaviour towards bitter tastants. Such a study case of the gustatory system of larval insects is interesting also from an applied perspective as these creatures eat up sizeable fractions of the global agricultural harvest.

## Materials and Methods

### Larvae

We use third-instar feeding-stage larvae from the Canton-Special wild-type strain, aged 5 days after egg laying. Flies are maintained on standard medium, in mass culture at 25°C, 60–70% relative humidity and a 14/10 hour light/ dark cycle. Before each experiment, we remove a spoonful of food medium from a food vial, collect the desired number of larvae, briefly rinse them in distilled water and start the experiment.

### Choice

The day before experiments, we prepare the Petri dishes (with 55 mm inner diameter; Sarstedt, Nümbrecht, Germany). We separate them into two halves with a piece of overhead transparency, fill one side with only 1% agarose (henceforth called PURE; electrophoresis grade; Roth, Karlsruhe, Germany) and the other side with 1% agarose added with quinine hemisulfate as a bitter tastant (henceforth called QUI; CAS: 6119-70-6; purity: 94%, Sigma-Aldrich, Seelze, Germany) at the respectively indicated concentrations; for the CONTROL condition, both sides of the Petri dish contain PURE. Shortly before the agarose is completely solidified, we remove the overhead transparency, and after appr. 10 minutes of cooling we cover the dishes with their lids and leave them at room temperature until the following day. Please note that we cannot exclude some level of diffusion of quinine into the PURE half of the Petri dish overnight, such that there might be a quinine gradient in the middle section of the Petri dish. Therefore we determined an appr. 1 cm wide middle zoe. Larvae inside this middle zone are included in the total number of equation (1) (see below). This procedure may underestimate QUI avoidance, but cannot lead to false-positive results.

Unless mentioned otherwise, we place 15 larvae in the middle of the dish and close the lid. The QUI-side is in half of the cases to the right and in the other half to the left, to balance for spurious effects of the experimental surround. We record the number of larvae on either side of the dish and calculate a gustatory preference index (PREF_Gustatory_) as:
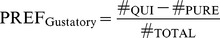
(1)


In this equation, # indicates the number of larvae on the respective side of the dish. Thus, PREF_Gustatory_ values are constrained between 1 and −1, positive values indicating a preference for QUI and negative values indicating aversion. These scores are taken at various time points after the animals are placed onto the dish (see Results for details).

### Feeding

To measure feeding behaviour on substrates containing QUI, we follow a procedure based on [21]: Ten larvae are placed on a 90 mm diameter Petri dish filled with either 1% agarose plus 30% red food dye (RU9805; http://www.backfun.de) (these are henceforth called PURE) or are filled with agarose, the food dye, plus the chosen concentration of QUI (see Results for details). The animals are allowed to feed on either of these respective substrates for 15min; then they are washed in tap water, transferred onto a Petri dish and placed on crushed ice for approximately 3min. Next, a haphazardly chosen individual larva from the PURE Petri dish is taken, briefly (approximately 2 s) put into boiling water and placed under a binocular coupled to a digital camera (Canon, model A650); we then place a haphazardly chosen companion larva from the QUI Petri dish next to the larva from the PURE dish and take a picture. For illumination we use a light table coupled to a cold-light source (Volpi, Schlieren, Switzerland). Using an imageJ-based, custom-written software, we determine for each larva the area of its body (Body) and the area of red colour (Red) in its gut. From these data, we calculate a Feeding Index (FI) as:
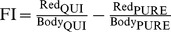
(2)


In this equation, Red_QUI_ indicates the area of red colour of an individual larva fed on QUI, and Body_QUI_ indicates the area of its body. Red_PURE_ indicates the area of red colour and Body_PURE_ the area of the body of the concomitantly photographed larva fed on PURE. Because the body area cannot possibly be smaller than the red-coloured area, FI values range from −1 to +1, with negative values indicating suppression of feeding by quinine and positive values indicating enhancement of feeding. Total body size did not differ between experimental groups (data not shown).

### Reinforcement

These experiments use Petri dishes of 90 mm diameter filled with either only 1% agarose (PURE) or with 1% agarose added with quinine (QUI) as negative reinforcer (−) at the concentrations indicated along the Results section.

Prior to experiments, odour containers are prepared: 10 µl of odour substance is filled into custom-made Teflon containers (5 mm inner diameter with a lid perforated with seven 0.5-mm diameter holes). As odour, we use *n*-amyl acetate (AM, 99% purity; Merk, Hohenbrunn, Germany), diluted 1∶50 in paraffin oil (Merk, Darmstadt, Hohenbrunn); in cases when no odour is presented, an empty container is used (EM) because the paraffin solvent is behaviourally ineffective for the larvae [21], [22]. Before the experiment starts, Petri dishes are covered with modified lids perforated in the centre by 15 holes with 1 mm diameter to improve aeration.

For training, we use a modified version of the one-odour reciprocal training regimen [22]. Thirty larvae are placed in the middle of a reinforcer-added dish with two odour containers on opposite sides (7 mm from the edges), both filled with AM. After 5 min, larvae are transferred onto an agarose-only dish with two empty containers, where they also spend 5 min. Three of these AM-/ EM training cycles are performed, each using fresh dishes. Along repetitions of the experiment, in half of the cases training starts with a reinforcer–added dish (AM-/ EM for all three training cycles) and in the other half with an agarose-only dish (EM/ AM- for all three training cycles).

Once training is completed, larvae are transferred to the middle of a quinine-containing Petri dish with two odour containers, this time filled with AM on one side and empty on the opposite side, to create a choice situation. Quinine is required during testing because aversive conditioned behaviour towards odour is a conditioned escape behaviour that is behaviourally expressed only if the test situation does indeed warrant escape (for a discussion see [23], [24]). After 3 min, the number of larvae on each side of the dish is noted and an olfactory preference (PREF) is calculated as:
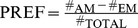
(3)


In this equation, # indicates the number of larvae observed on the respective side of the dish. PREF values thus can range between 1 and −1, positive values indicating preference for and negative values avoidance of AM.

For each group of larvae trained AM-/ EM (or EM/ AM-, respectively), a second group is trained reciprocally, i.e. by unpaired presentations of odour and quinine: AM/ EM- (or EM-/ AM, respectively). Aversive associative learning shall result in a stronger avoidance for AM after AM-/ EM training than after AM/ EM- training. This difference is quantified by the performance index (PI) as:
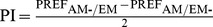
(4)


Here, PREF_AM-/ EM_ is the AM preference of the AM-/ EM trained group and PREF_AM/ EM-_ is that of the reciprocally trained AM/ EM- group (or of the respectively other training trial sequence). This PI is a measure of associative learning because it measures the difference in preference between two groups trained reciprocally, but otherwise treated the same (i.e. with respect to handling, exposure to odours and exposure to the reinforcer). PI values thus range between 1 and −1, positive values indicating conditioned approach towards the reinforcer-paired odour (appetitive learning) and negative values indicating conditioned avoidance of the reinforced odour (aversive learning).

### Statistical analyses

All statistical analyses are performed with Statistica on a PC. Preference values, feeding indices and performance indices are compared across multiple groups with Kruskal-Wallis tests. For subsequent pair-wise comparisons, Mann-Whitney U-tests are used. To test whether values of a given group differ from zero, we use one-sample sign tests. When multiple one-sample sign tests, Kruskal-Wallis tests, or Mann-Whitney U-tests are performed within one experiment, we adjust significance levels by a Bonferroni correction to keep the experiment-wide error rate at 5%. This is done by dividing the critical *P* value of 0.05 by the number of tests. We present our data as box plots which represent the median as the middle line and 25%/75% and 10%/90% as box boundaries and whiskers, respectively.

## Results

### Choice

First, we seek a suitable assay duration for testing bitter avoidance of experimentally naïve larvae; this is warranted because here, following the approach of Schipanski and colleagues analyzing sugar-initiated behaviour [25] we use assay plates with smaller diameter (approximately 55 mm) than in previous studies on quinine-related behaviour (approximately 90 mm) [21]. We restrict ourselves to a total observation time of 8 min because after that time point the larvae begin to dig into the substrate or to crawl up the side walls of the Petri dish (not shown); we chose 5 mM of QUI because this concentration had been used in previous work and because higher concentrations of QUI, without acidification, show crystalization of QUI in the agarose dishes. Thus, we allow the larvae to choose between pure agarose (PURE) and agarose added with 5 mM QUI and recurrently score for their choice after 1, 2, 4, and 8 min.

**Figure 2:**
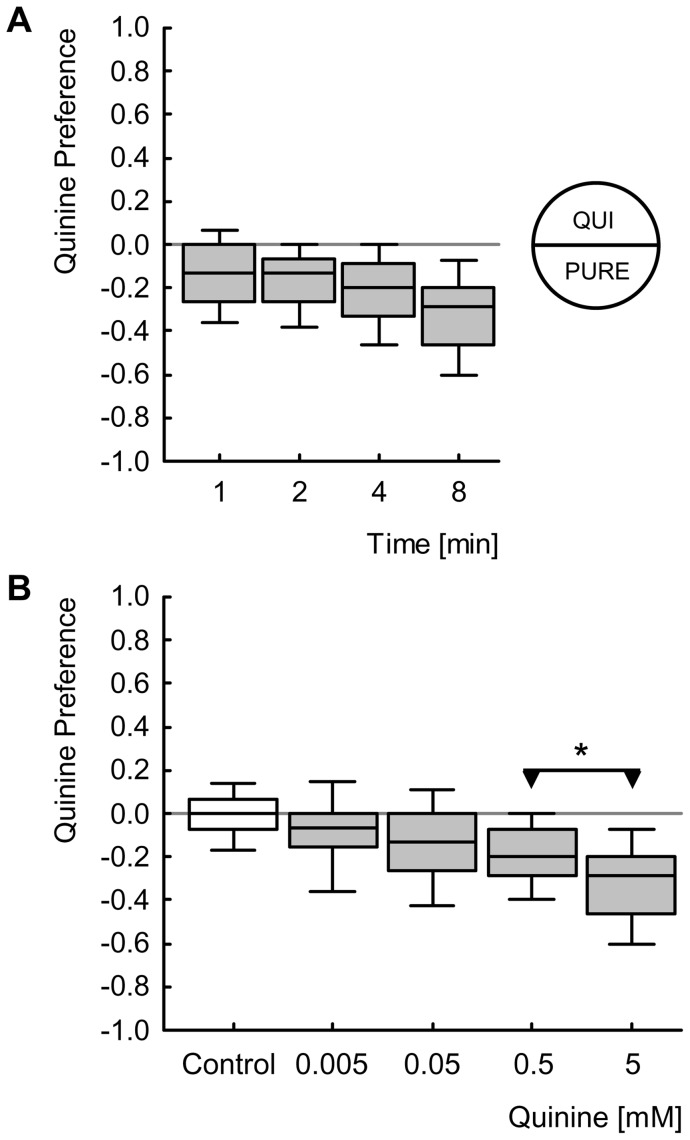
Choice. (A) Larvae are allowed to chose between one side of a Petri dish that contains agarose added with 5 mM quinine (QUI), and pure agarose (PURE) on the other side. A gustatory preference (PREF_Gustatory_) is calculated at different time points after the experiment has started. Negative PREF_Gustatory_ values indicate avoidance of QUI which is statistically significant already from 1 min on. Based on these results we chose 8 min as time point of scoring choice in subsequent experiments, as for this time point avoidance was apparently strongest. (B) Gustatory preference either between both sides of a Petri dish filled with agarose-only (CONTROL) or between a QUI- and a PURE-filled side of a split Petri dish, at the indicated concentrations of QUI. Scores are taken after 8 min. At concentrations from 0.005 mM to 5 mM QUI is avoided by the larvae. Avoidance differs between the two highest concentrations of QUI, thus arguing that the point of asymptote should be above 5 mM. Shading of the boxes indicates significant differences from chance behaviour (i.e. zero, one-sample sign tests) (A) *P*<0.05/4; (B) *P*<0.05/7; keeping the experiment-wide error rate at 5% (i.e. Bonferroni correction). Labelling of * refers to *P*<0.05 in a Mann-Whitney U-test. Box plots represent the median as the middle line and 25%/75% and 10%/90% as box boundaries and whiskers, respectively. Sample sizes: (A) N = 101; (B) from left to right N = 110, 150, 120, 95, 101.

We observe avoidance of 5 mM QUI beginning already from 1 min after assay-onset (Fig. 2A; one-sample sign tests *P*<0.05/4 for all time points; sample size N = 101). We chose 8 min as observation time for all subsequent analyses, as for this time point avoidance was apparently strongest, albeit not yet asymptotic. We note that we have previously found that when using fructose, sucrose or trehalose rather than QUI, asymptotic preference is found after less than 8 min [25], arguing that from the motor side and the spatial layout of the assay the allowed time is enough for the larvae to behaviourally express their preference, if they have any.

We next asked whether, when assayed after 8 min, the avoidance of QUI depends on its concentration. Using either CONTROL Petri dishes that contain PURE agarose on both sides or Petri dishes with one side including QUI and the other side PURE, we probe for QUI avoidance in a range from 5 mM down to 0.005 mM. Obviously, the concentration of QUI matters for larval avoidance behaviour (Fig. 2B; Kruskal-Wallis test: *P*<0.05, H = 150, df = 4, sample sizes N = 110, 150, 120, 95, 101). When both sides of the Petri dish lack QUI, the larvae distribute equally between both sides (Fig. 2B; CONTROL one-sample sign test: *P* = 0.36; sample size as above). However, already for the lowest concentration (0.005 mM) larvae show a weak yet, given the very large sample size, significant avoidance of QUI (Fig. 2B; one-sample sign tests: *P<*0.05/5; sample size as above) (for the respective other concentrations: *P*<0.05/5 as well; sample sizes as above). Notably, values for the two highest concentrations still do differ from each other (Fig. 2B; Mann-Whitney U-test; U = 2969; *P*<0.05; sample sizes as above), arguing that the point of asymptote should be above 0.5 mM, and thus beyond the range of concentrations that can be used without acidifying the agarose (see above).

### Feeding

**Figure 3:**
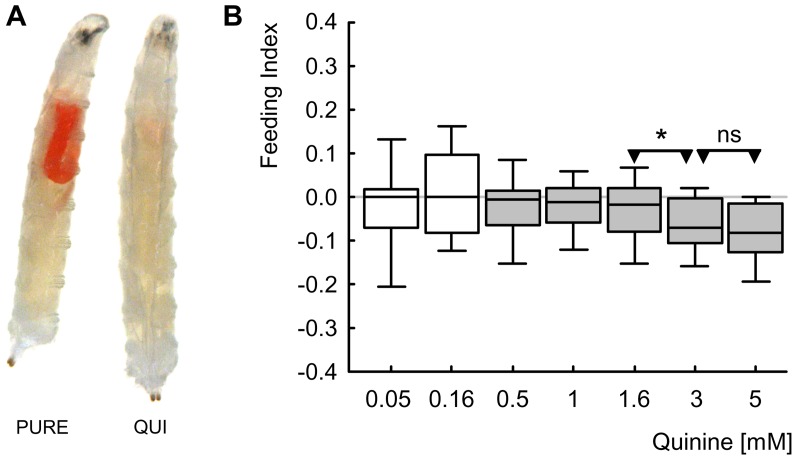
Feeding. (A) Larvae are allowed to feed on either a red-dyed, PURE Petri dish (left) or on a red-dyed, 5 mM QUI-containing Petri dish (right), for 15 minutes. Using a digital camera, a picture is taken and the area of red colour in individual larvae (indicating the amount eaten) as well as the area size of the whole body is determined for the calculation of the Feeding Index. (B) Feeding Indices of larvae fed on QUI in a concentration range between 0.05 mM and 5 mM. Positive Feeding Index values (FI) indicate that larvae eat more on a QUI than on a PURE Petri dish, negative scores indicate QUI-induced suppression of feeding. Larvae show feeding suppression for QUI concentrations in a range from 0.5 mM to 5 mM. Please mind the truncated axis. Shading of the boxes indicates significant differences from chance behaviour (i.e. zero, one-sample sign tests) (*P*<0.05/7, keeping the experiment-wide error rate at 5% [i.e. Bonferroni correction]); labelling of * or ns refers to *P*<0.05/2 or *P*>0.05/2 in Mann-Whitney U-tests. Box plots represent the median as the middle line and 25%/75% and 10%/90% as box boundaries and whiskers, respectively. Sample sizes: from left to right N = 90, 70, 200, 119, 147, 70, 162.

In the next experiment we seek to confirm the previously reported suppressing effect of 5 mM QUI on larval feeding [21] (Fig. 3A) and to extend that finding with respect to its dose-effect characteristic (Fig. 3B; Kruskal-Wallis test: *P*<0.05, H = 75.2, df = 6, sample sizes N = 90, 70, 200, 119, 147, 70, 162). Concentrations of QUI ranging from 0.5 mM to 5 mM lead to feeding suppression (Fig. 3B; one-sample-sign tests: *P*<0.05/7, sample sizes see above); the two lowest QUI concentrations (0.05 mM and 0.16 mM), however, leave feeding unaffected (Fig. 3B; one-sample-sign tests: *P*>0.05/7, sample sizes see above). Feeding Indices do not differ between the two highest concentrations (Fig. 3B; Mann-Whitney U-test: *P*>0.05/2, U = 5017, sample sizes as above), arguing that the asymptote of feeding suppression is reached at a concentration of lower than 3 mM; as, in turn, feeding suppression for 3 mM is stronger than for the next lower concentration (Fig. 3B; Mann-Whitney U-test: *P*<0.05/2, U = 3825, sample sizes as above), the concentration of asymptote should be higher than 1.6 mM.

### Reinforcement

**Figure 4:**
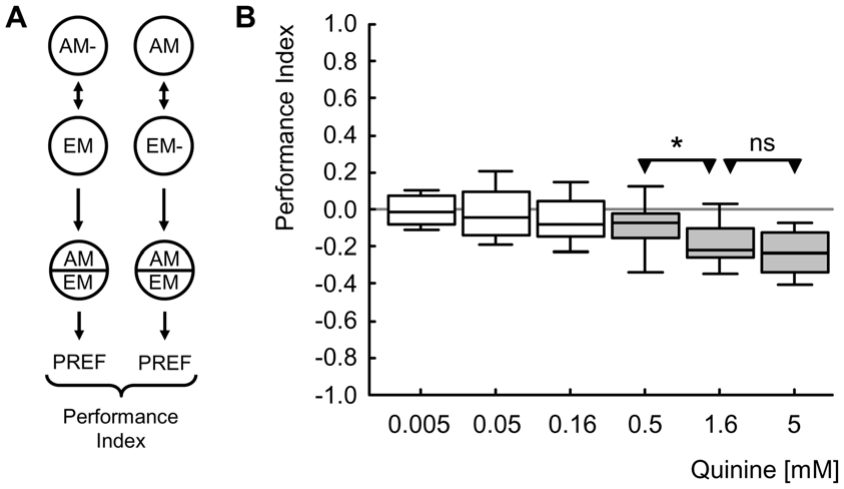
Reinforcement. (A) Larvae are trained such that one group of larvae receives *n*-amylacetate while crawling on a QUI-containing Petri dish, whereas an empty odour container is presented in the absence of the reinforcer (i.e., AM-/EM training; left). Another group is trained reciprocally, i.e. with unpaired presentations of AM and reinforcement (AM/EM-; right) (note that for half of the cases the sequence of trials is as indicated; for the other half, sequences are reversed: EM/AM- and EM-/AM, respectively). After three such training cycles, larvae from both groups are tested on a QUI-containing Petri dish for their preference between AM and EM in a choice situation (for a documentation of these preference scores, see Figure S1). Associative learning is revealed by lower preference scores for AM in the group trained AM-/EM than in the reciprocally trained AM/EM- group. This difference is quantified by the displayed Performance Index (PI), such that negative PI values indicate conditioned avoidance. (B) The strength of QUI reinforcement depends on its concentration. In the range of concentrations tested (from 0.005 mM to 5 mM) the three highest concentrations support learning, but the three lowest concentrations do not. We find that QUI reinforcement reaches asymptote between 0.5 mM and 1.61 mM. The shading of the boxes indicates significant differences from zero, i.e. from chance behaviour (*P*<0.05/6 in one-sample sign tests, keeping the experiment-wide error rate at 5% [i.e. Bonferroni correction]); labelling of * or ns refer to *P*<0.05/2 or *P*>0.05/2 in Mann-Whitney U-tests. Box plots represent the median as the middle line and 25%/75% and 10%/90% as box boundaries and whiskers, respectively. Sample sizes are from left to right N = 13, 46, 29, 49, 29, 76).

Next, we probe QUI-induced aversive learning for its dose-dependency. We train larvae with either of six concentrations of QUI (5 mM, 1.61 mM, 0.5 mM, 0.16 mM, 0.05 mM, 0.005 mM) and compare these concentrations in terms of their reinforcement potency (Fig. 4; for the corresponding preference scores see Fig. S1). These different QUI concentrations differ in terms of the associative Performance Index they support (Fig. 4; Kruskal-Wallis test: *P*<0.05, H = 56.5, df = 5, sample sizes N = 13, 46, 29, 49, 29, 76), such that the three highest concentrations (5 mM, 1.61 mM and 0.5 mM) support significant aversive learning (Fig. 4; one-sample sign-tests: *P*<0.05/6, sample sizes as above), whereas the three lowest concentrations (0.16 mM, 0.05 mM and 0.005 mM) do not (Fig. 4; one-sample sign-tests: *P>*0.05/6, sample size as above). When compared between the two highest concentrations, Performance Indices do not differ (Fig. 4; Mann-Whitney U-test: *P*>0.05/2, U = 988, sample sizes as above), arguing that the reinforcement potency of QUI reaches asymptote at a concentration lower than 1.61 mM; as, in turn, Performance Indices for the second-highest concentration are stronger than for the middle concentration (Fig. 4; Mann-Whitney U-test: *P*<0.05/2, U = 434, sample sizes as above), the point of asymptote should be higher than 0.50 mM.

### Comparing dose-effect functions for feeding and reinforcement

**Figure 5:**
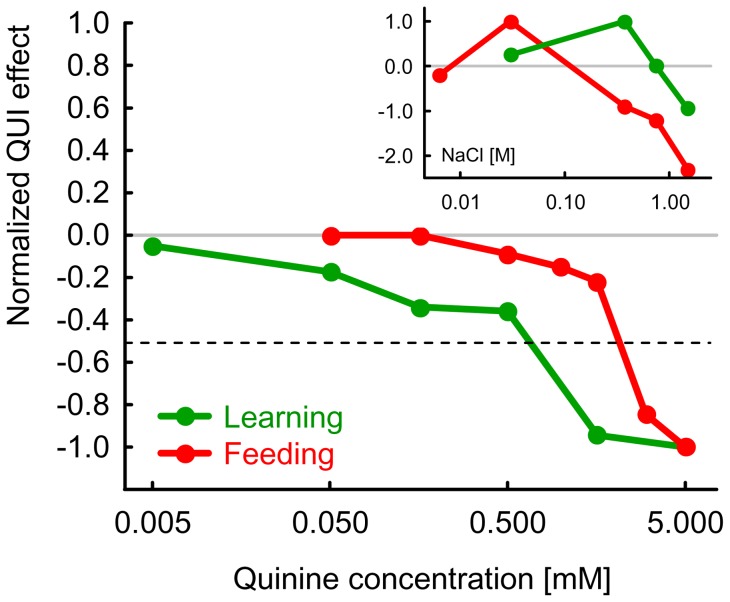
Summary of the dose-effect functions of feeding and learning. We divide the median values for each quinine concentration by the lowest median value found within one experiment (and multiply by −1), thus yielding the displayed normalized scores (green: Learning; red: Feeding). The half-maximal effect of QUI on feeding appears to require a concentration of QUI that is almost one order of magnitude higher than for learning. As for choice the maximal effect can for practical reasons not be determined (see Discussion), a similar display for choice would insinuate a too low concentration as “half-maximal” (see Fig. S2), and is therefore not included. The inset uses data from [26] to display the dose-effect functions of feeding and learning for sodium chloride for comparison. Please note that changing the concentration of sodium chloride in the rearing-food may alter the balance between its appetitive and aversive effects [36].

Given that for feeding and for the reinforcement function the concentration at which QUI exerts an asymptotic effect, and thus the concentration at which the effect is half-maximal, could be determined (Figs. 3,4), we decided to provide an overview of the these results as follows: We divide the median Feeding Index for each concentration by the strongest median Feeding Index found (i.e. by the one for 5 mM QUI) (and multiply by −1), thus yielding the normalized Feeding scores displayed in Figure 5 (red). The data from the leaning experiment (Fig. 4) were treated accordingly, yielding the normalized Learning scores displayed in Figure 5 (green).

## Discussion

In the present experiments on feeding and learning the concentrations at which the behavioural effects of quinine reach asymptote, and hence those concentrations which yield half-maximal effects, could be determined (Fig. 5); for choice, however, such asymptote is not reached at the highest concentration used in the current experiments (Fig. S2). As mentioned, that concentration (5 mM QUI) is the highest one that can be used without acidification; however, such acidification is not warranted because it introduces confounding gustatory and olfactory cues. In this context, one may wonder what the acidity of those microhabitats is in which the larvae encounter QUI in the wild, and/or whether the more natural role of QUI in choice behaviour is a modulation of sugar processing (see below). Still, from a practical point of view, another option to detect the asymptote of QUI choice behaviour would have been to allow the larvae more time for choice; however, as mentioned above we observed that for longer assay durations the larvae start digging into the agarose. Thus, with the present experiments we cannot determine the “true” asymptote of quinine choice behaviour. Therefore we restrict the below discussion to feeding and learning.

### Comparing the dose-effect functions for feeding and learning

For feeding, a half-maximal effect appears to require a concentration of QUI that is substantially higher than for learning (Fig. 5). Why is this so?

Potentially, these differences could be due to differences in sensitivity of the respective behavioural assays: For example, feeding as the primary occupation of the larvae may be particularly hard to suppress. Also, the learning assay measures the behaviour of populations of larvae, while the feeding assay considers individual behaviour, potentially making decreases in feeding more difficult to detect. However, when using NaCl rather than QUI, Niewalda and colleagues [26] found that the dose-effect function for feeding modulation is shifted by one order of magnitude towards lower concentrations as compared to learning (see inset of Fig. 5), and Schipanski and colleagues [25] reached the same conclusion regarding fructose/sucrose, lending no support to the notion that the feeding assay *per se* were, for whatever reason, different in sensitivity from the learning experiment.

**Figure 6:**
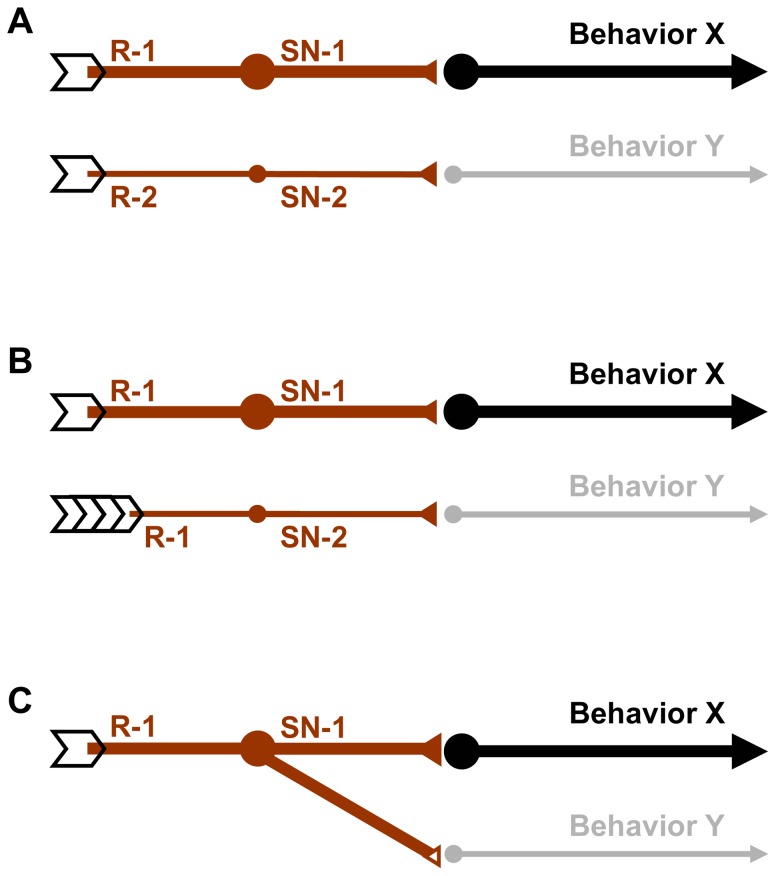
Accounting for differences in dose-effect functions for different behaviours. Schematic sketches of how different dose-effect characteristics of bitter processing with respect to different behaviours could come about. (A) Different sensory neurons (SN-1, SN-2) steering different behaviours (Behaviour X and Behaviour Y) may differ in the receptors expressed (R-1, R-2, with different ligand sensitivity), or (B) in the sensory-organ origin of their host sensory neurons, such that one of them is less exposed to QUI because of e.g. dilution by saliva. (C) One and the same subset of sensory neurons may diverge to form synaptic connections of different gain to downstream neurons. In all three cases, a particular QUI concentration would be more powerful to elicit behaviour X than behaviour Y.

As an alternative explanation, feeding and the reinforcing effect of QUI may rely on distinct processing streams, differing in their dose-effect characteristics (Fig. 6):

- Suppose different sets of receptor molecules (R-1 and R-2) were expressed in different sensory neurons (SN-1 and SN-2), differing in their dose-effect characteristics (indicated by the thickness of the lines). Thus, the behaviours respectively steered by them will follow these respective tunings (Fig. 6A).
- Suppose pharyngeal sensory neurons were involved in organizing feeding behaviour but externally located sensory neurons were responsible for triggering reinforcement (Fig. 6B). As the ingested substrate will be diluted by saliva, the actual concentration of QUI in the pharynx will be lower at the internal taste organs, requiring the experimenter to use higher concentrations to reach full effect for feeding. While in principle conceivable, we note that regarding sodium chloride and fructose/sucrose the argument would need to be made just the other way around (see above).
- Lastly, a given sensory neuron may form connections towards downstream neurons at different gains. For example, downstream neurons suppressing feeding may require stronger input from sensory neurons than neurons mediating internal reinforcement (Fig. 6C).

In principle, the relatively fragmented knowledge of the larval contact chemosensory system allows for either of these three interpretations: Candidate larval gustatory sensory neurons are located in both external and internal sense organs (Fig. 1), both of which include non-gustatory sensory neurons as well (whether the so-called p-es organs in the ventral pits [12] on the thoracic and/or abdominal segments contribute to processing of classical tastants is presently unknown). The external ones are the terminal (26 gustatory sensory neurons) and the ventral organ (7) and the bulge of the dorsal organ (9). The internal sensillae are located along the pharynx and are organized into dorsal, ventral and posterior pharyngeal sensillae (16; 15; 6) [6], [7]. All these gustatory sensory neurons project to the subesophageal ganglion (SEG) (Fig. 1) [6], [7]. Importantly, in larval and in adult *Drosophila*, target regions of gustatory sensory neurons within the SEG seem to depend both on the location of the peripheral taste organs from which they originate and on their behavioural function [6], [7], [18].

On the molecular level, primary bitter as well as sugar taste processing in larval and adult *Drosophila* at least partially relies on the *Gr*- and possibly the *Ir*- [18] and/or *Trp*-family [27] of receptors: In adult *Drosophila*, distinct sets of gustatory sensory neurons express putative sugar (*Gr5a*) or bitter (*Gr66a*) receptor genes [28], [29]. Driving *Gr5a*- or *Gr66a*-positive neurons is sufficient to induce attraction or avoidance, respectively [29]. However, bitter tastants can not only activate the neurons that express a respectively tuned receptor, but can also inhibit sugar receptor-expressing cells as well as water-sensitive cells when presented in mixture with sugars or in aqueous solution [30]. Consistent with this triple effect of bitter tastants, disabling the *Gr66a*-positive neurons reduces, but does not abolish, the capacity of bitter tastants to inhibit proboscis extension towards sugars [28]. Notably, bitter-sensitive gustatory neurons typically seem to express more than one *Gr* gene [17], [31]–[34].

Regarding the larva, *Gr66a* is expressed in the larval terminal organ and likely also in gustatory neurons along the pharynx [7], [35]. Like in adults, all gustatory cells of the terminal organ expressing *Gr66a* also express *Gr33a*. In addition, 15 other *Grs*, including *Gr32a*, are co-expressed with *Gr66a* in partially overlapping sets of cells [35]. Also, driving *Gr66a*-expressing neurons induces larval avoidance behaviour [7], but strikingly no sugar-sensitive *Gr* has yet been found in larvae [35]. A similarly detailed analysis of *Irs* in the larva is not yet available.

In adults it has been reported that there exist different types of taste sensillae responding to different subsets of bitter substances [30], [34]. For example, after genetic ablation of the TrpA1 channel, behaviour of adults towards aristolochic acid was reduced, without affecting behaviour regarding other bitter substances like quinine, denatonium, berberine or strychinine [27].

To summarize, it appears reasonable to reckon with substantial diversity between bitter-sensitive sensory neurons, including differences in their receptor complements, ligand profiles, dose-effect characteristics, connectivity to downstream neurons and behavioural roles. It should be interesting to see whether this diversity will eventually be seen as drastic enough to abandon the bitter-category altogether. In any event, for quinine as an example this study provides a first systematic step to investigate how the gustatory system in the larva is organized to support different kinds of behaviour towards a bitter tastant.

## Supporting Information

Figure S1 **Preference scores of all groups of larvae from the reinforcement experiment.** Animals receive either an AM-/ EM training or an AM/ EM- training and are subsequently tested on a QUI-containing Petri dish for their choice between AM and EM, as indicated in the sketches below the boxes. Differences in preference scores between two corresponding reciprocally trained groups (e.g. the two right-most panels) result in Performance Index (PI) scores different from zero (see Fig. 4, right-most panel). Note that in half of the cases the sequence of training trials is as indicated (in the right-most panel e.g. AM/ EM-), but in the other half it is reversed (e.g. EM-/ AM). The stippled line represents the median of the pooled six left-most boxes, showing that for higher concentrations of QUI paired and unpaired presentations of AM and QUI result in decreases and increases in preferences scores, respectively. The shading of the boxes indicates significant differences from zero in one-sample sign tests, i.e. from chance behaviour (*P<*0.05/12, keeping the experiment-wide error rate at 5% [i.e. Bonferroni correction]); labelling of * or NS refer to *P*<0.05/6 or *P*>0.05/6 in Mann-Whitney U-tests. Box plots represent the median as the middle line and 25%/75% and 10%/90% as box boundaries and whiskers, respectively. Sample sizes are from left to right N = 13, 13, 46, 46, 29, 29, 49, 49, 29, 29, 76, 76).
(TIF)

Figure S2 **Dose-effect function of choice behaviour.** We divide the median choice values for each quinine concentration by the lowest median value found (and multiply by −1), thus yielding the displayed normalized choice scores. The dose-effect curve seems to be linear, because for practical reasons a plateau in choice scores cannot be determined (see Discussion).
(TIF)

## References

[1] Hammer M (1997). The neural basis of associative reward learning in honeybees.. Trends Neurosci.

[2] Hammer M, Menzel R (1998). Multiple sites of associative odor learning as revealed by local brain microinjections of octopamine in honeybees.. Learn Mem.

[3] Menzel R, Heyne A, Kinzel C, Gerber B, Fiala A (1999). Pharmacological dissociation between the reinforcing, sensitizing, and response-releasing functions of reward in honeybee classical conditioning.. Behav Neurosci.

[4] de Araujo IE, Oliveira-Maia AJ, Sotnikova TD, Gainetdinov RR, Caron MG (2008). Food reward in the absence of taste receptor signaling.. Neuron.

[5] Heimbeck G, Bugnon V, Gendre N, Haberlin C, Stocker RF (1999). Smell and taste perception in *Drosophila melanogaster* larva: toxin expression studies in chemosensory neurons.. J Neurosci.

[6] Python F, Stocker RF (2002). Adult-like complexity of the larval antennal lobe of *D. melanogaster* despite markedly low numbers of odorant receptor neurons.. J Comp Neurol.

[7] Colomb J, Grillenzoni N, Ramaekers A, Stocker RF (2007). Architecture of the primary taste center of *Drosophila melanogaster* larvae.. J Comp Neurol.

[8] Gerber B, Stocker RF (2007). The *Drosophila* larva as a model for studying chemosensation and chemosensory learning: a review.. Chem Senses.

[9] Melcher C, Bader R, Pankratz MJ (2007). Amino acids, taste circuits, and feeding behavior in *Drosophila*: towards understanding the psychology of feeding in flies and man.. J Endocrinol.

[10] Stocker RF (2008). Design of the larval chemosensory system.. Adv Exp Med Biol.

[11] Gerber B, Stocker RF, Tanimura T, Thum AS (2009). Smelling, Tasting, Learning: *Drosophila* as a Study Case.. Results Probl Cell Differ.

[12] Cobb M, Scott K, Pankratz M (2009). Gustation in Drosophila melanogaster.. SEB Exp Biol Ser.

[13] Stocker RF (1994). The organization of the chemosensory system in *Drosophila melanogaster*: a review.. Cell and Tissue Research.

[14] Ishimoto H, Tanimura T (2004). Molecular neurophysiology of taste in *Drosophila*.. Cell Mol Life Sci.

[15] Ebbs ML, Amrein H (2007). Taste and pheromone perception in the fruit fly *Drosophila melanogaster*.. Pflugers Arch.

[16] Vosshall LB, Stocker RF (2007). Molecular architecture of smell and taste in *Drosophila*.. Annu Rev Neurosci.

[17] Montell C (2009). A taste of the *Drosophila* gustatory receptors.. Curr Opin Neurobiol.

[18] Isono K, Morita H (2010). Molecular and cellular designs of insect taste receptor system.. Front Cell Neurosci.

[19] Clyne PJ, Warr CG, Carlson JR (2000). Candidate taste receptors in *Drosophila*.. Science.

[20] Benton R, Vannice KS, Gomez-Diaz C, Vosshall LB (2009). Variant ionotropic glutamate receptors as chemosensory receptors in *Drosophila*.. Cell.

[21] Hendel T, Michels B, Neuser K, Schipanski A, Kaun K (2005). The carrot, not the stick: appetitive rather than aversive gustatory stimuli support associative olfactory learning in individually assayed *Drosophila* larvae.. J Comp Physiol A Neuroethol Sens Neural Behav Physiol.

[22] Saumweber T, Husse J, Gerber B (2011). Innate attractiveness and associative learnability of odors can be dissociated in larval Drosophila.. Chem Senses.

[23] Gerber B, Hendel T (2006). Outcome expectations drive learned behaviour in larval *Drosophila*.. Proc Biol Sci.

[24] Schleyer M, Saumweber T, Nahrendorf W, Fischer B, von Alpen D (2011). A behavior-based circuit model of how outcome expectations organize learned behavior in larval Drosophila.. Learn Mem.

[25] Schipanski A, Yarali A, Niewalda T, Gerber B (2008). Behavioral analyses of sugar processing in choice, feeding, and learning in larval *Drosophila*.. Chem Senses.

[26] Niewalda T, Singhal N, Fiala A, Saumweber T, Wegener S (2008). Salt processing in larval *Drosophila*: choice, feeding, and learning shift from appetitive to aversive in a concentration-dependent way.. Chem Senses.

[27] Kim SH, Lee Y, Akitake B, Woodward OM, Guggino WB (2011). Drosophila TRPA1 channel mediates chemical avoidance in gustatory receptor neurons.. Proc Natl Acad Sci U S A.

[28] Wang Z, Singhvi A, Kong P, Scott K (2004). Taste representations in the *Drosophila* brain.. Cell.

[29] Marella S, Fischler W, Kong P, Asgarian S, Rueckert E (2006). Imaging taste responses in the fly brain reveals a functional map of taste category and behavior.. Neuron.

[30] Meunier N, Marion-Poll F, Rospars JP, Tanimura T (2003). Peripheral coding of bitter taste in *Drosophila*.. J Neurobiol.

[31] Lee Y, Moon SJ, Montell C (2009). Multiple gustatory receptors required for the caffeine response in *Drosophila*.. Proc Natl Acad Sci U S A.

[32] Moon SJ, Lee Y, Jiao Y, Montell C (2009). A *Drosophila* gustatory receptor essential for aversive taste and inhibiting male-to-male courtship.. Curr Biol.

[33] Miyamoto T, Amrein H (2008). Suppression of male courtship by a *Drosophila* pheromone receptor.. Nat Neurosci.

[34] Weiss LA, Dahanukar A, Kwon JY, Banerjee D, Carlson JR (2011). The molecular and cellular basis of bitter taste in Drosophila.. Neuron.

[35] Kwon JY, Dahanukar A, Weiss LA, Carlson JR (2011). Molecular and cellular organization of the taste system in the Drosophila larva.. J Neurosci.

[36] Russell C, Wessnitzer J, Young JM, Armstrong JD, Webb B (2011). Dietary salt levels affect salt preference and learning in larval Drosophila.. PLoS One.

