# Supplementary figures and images for: Behavioural Analyses of Quinine Processing in Choice, Feeding and Learning of Larval Drosophila
Source: PLoS One. 2012 Jul 10;7(7):e40525. doi: 10.1371/journal.pone.0040525 (PMC3393658; doi:10.1371/journal.pone.0040525)

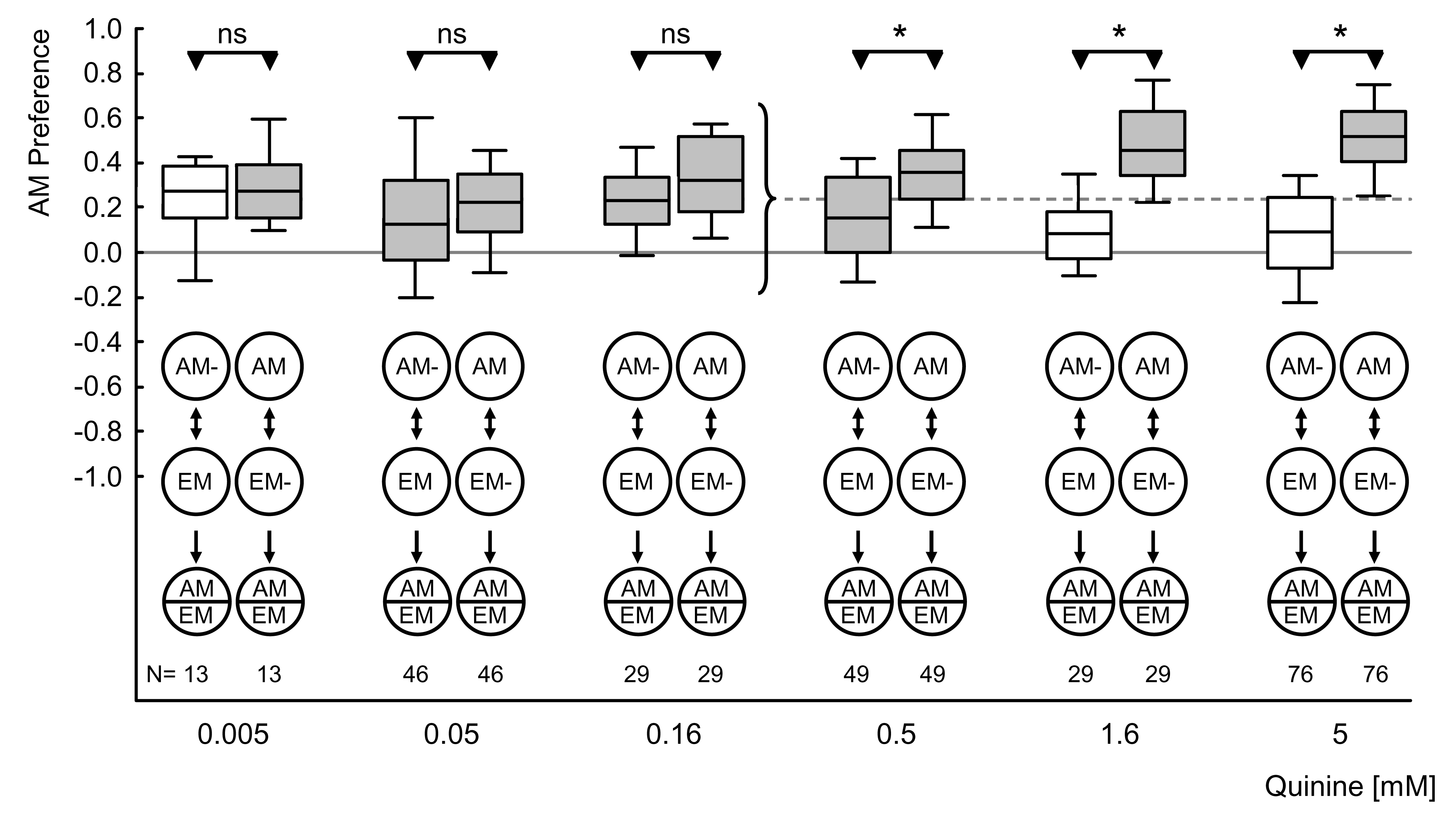

Supplement: Figure S1 — Preference scores of all groups of larvae from the reinforcement experiment. Animals receive either an AM-/ EM training or an AM/ EM- training and are subsequently tested on a QUI-containing Petri dish for their choice between AM and EM, as indicated in the sketches below the boxes. Differences in preference scores between two corresponding reciprocally trained groups (e.g. the two right-most panels) result in Performance Index (PI) scores different from zero (see Fig. 4, right-most panel). Note that in half of the cases the sequence of training trials is as indicated (in the right-most panel e.g. AM/ EM-), but in the other half it is reversed (e.g. EM-/ AM). The stippled line represents the median of the pooled six left-most boxes, showing that for higher concentrations of QUI paired and unpaired presentations of AM and QUI result in decreases and increases in preferences scores, respectively. The shading of the boxes indicates significant differences from zero in one-sample sign tests, i.e. from chance behaviour (P<0.05/12, keeping the experiment-wide error rate at 5% [i.e. Bonferroni correction]); labelling of * or NS refer to P<0.05/6 or P>0.05/6 in Mann-Whitney U-tests. Box plots represent the median as the middle line and 25%/75% and 10%/90% as box boundaries and whiskers, respectively. Sample sizes are from left to right N = 13, 13, 46, 46, 29, 29, 49, 49, 29, 29, 76, 76). (TIF) [file pone.0040525.s001.tif]

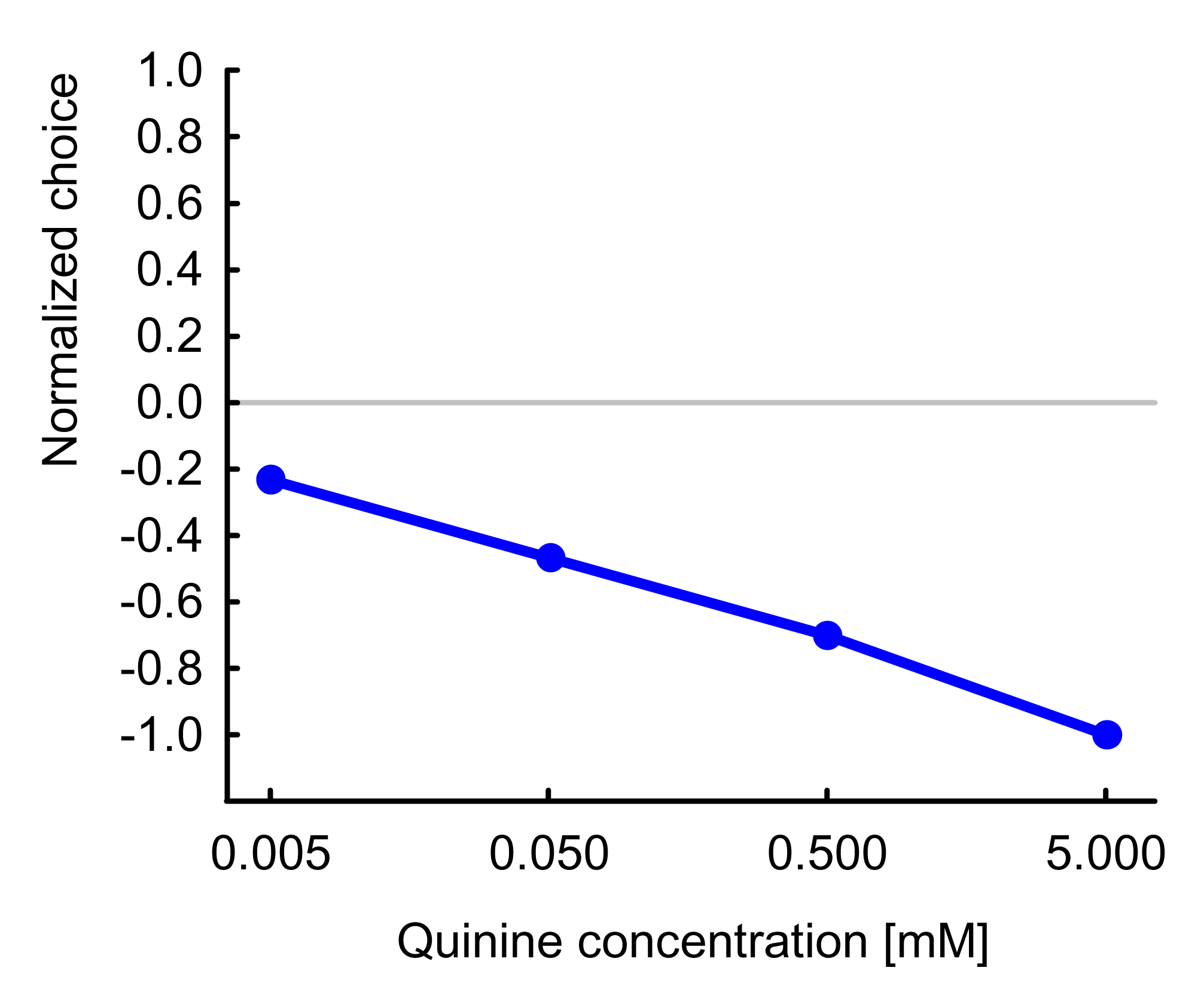

Supplement: Figure S2 — Dose-effect function of choice behaviour. We divide the median choice values for each quinine concentration by the lowest median value found (and multiply by −1), thus yielding the displayed normalized choice scores. The dose-effect curve seems to be linear, because for practical reasons a plateau in choice scores cannot be determined (see Discussion). (TIF) [file pone.0040525.s002.tif]
